# DelaySSA: stochastic simulation of biochemical systems and gene regulatory networks with or without time delays

**DOI:** 10.1371/journal.pcbi.1012919

**Published:** 2025-04-08

**Authors:** Ziyan Jin, Xinyi Zhou, Zhaoyuan Fang

**Affiliations:** 1 Department of Colorectal Surgery and Oncology of the Second Affiliated Hospital, and Centre of Biomedical Systems and Informatics of Zhejiang University-University of Edinburgh Institute (ZJU-UoE Institute), Zhejiang University School of Medicine, Zhejiang University, Hangzhou, China; 2 State Key Laboratory of Bioreactor Engineering, East China University of Science and Technology, Shanghai, China; 3 Edinburgh Medical School, College of Medicine and Veterinary Medicine, The University of Edinburgh, Edinburgh, United Kingdom; 4 Key Laboratory of Cancer Prevention and Intervention, China National Ministry of Education, Hangzhou, China; 5 Biomedical and Health Translational Research Center of Zhejiang Province, Haining, China; Fudan University, CHINA

## Abstract

Stochastic Simulation Algorithm (SSA) is crucial for modeling biochemical reactions and gene regulatory networks. Traditional SSA is characterized by Markovian property and cannot naturally model systems with time delays. Several algorithms have already been designed to handle delayed reactions, yet few easy-to-use implementations exist. To address these challenges, we have developed DelaySSA, an R package that implements currently available algorithms for SSA with or without delays. Meanwhile, we also provided Matlab and Python versions to support wider applications. We demonstrated its accuracy and validity by simulating two classical models: the Bursty model and Refractory model. We then tested its capability to simulate the RNA Velocity model, where it successfully reproduced both the up- and down-regulation stages in the phase portrait. Finally, we extended its application to simulate a gene regulatory network of lung cancer adeno-to-squamous transition (AST) and qualitatively analyzed its bistability behavior by approximating the Waddington’s landscape. Modeling the therapeutic intervention of a SOX2 degrader as a delayed degradation reaction, AST is effectively blocked and reprogrammed back to the adenocarcinoma state, providing a useful clue for targeting drug-resistant AST in the future. Taken together, DelaySSA is a powerful and easy-to-use software suite, facilitating accurate modeling of various kinds of biological systems and broadening the scope of stochastic simulations in systems biology.

## Introduction

Stochastic Simulation Algorithm (SSA) has become a widely used tool in modeling biochemical reactions [1]. This algorithm directly simulates the temporal evolution of a spatially homogeneous system of molecular species undergoing reactions, providing exact time trajectories that reflect the stochastic and fluctuating nature of biochemical processes [2–5].

Importantly, SSA is capable of simulating many cellular and molecular models that are recently under extensive studies. For instance, one class of models is the molecular mechanisms driving gene expression state dynamics of individual cells, which have become a highly influential and fruitful field in single-cell studies of development and diseases [6–9]. Another example class of models includes the molecular driver networks underlying various biological processes, such as the lung cancer adeno-to-squamous (AST) lineage transition models explaining targeted-therapy-related drug resistance [10–12]. Simulating these models with SSA is expected to produce results that are consistent with those obtained using ordinary differential equation (ODE) methods, while offering a more natural examination of the fluctuations inherent to these complex biomedical systems.

One of the characteristics of SSA is the Markovian property, with the future state depending on the present state only and not on the past. Thus, the current state fully specifies the probability of a reaction occurring in the very next infinitesimal time interval [13,14]. This Markovian assumption is a simplified modeling of dynamical processes for efficient computation. However, such simplification also leads to its incapability of modeling processes where historical events influencing the timing of the future events. In biological systems, delays are widespread. For example, gene expression processes such as transcription and translation inherently do require time to complete [15–18].

To take into account those delay effects, several methods have been developed to extend the standard Gillespie algorithm by simulating delayed reactions [19–21]. These algorithms are effective in handling delays, though with additional complexity introduced and technically being more difficult to understand and apply, particularly for researchers without computational expertise [22]. On the other hand, currently available software packages mainly focus on simulate SSA reactions without delay effects [23,24] and very few efforts have been devoted to implementing delayed simulations [25]. In order to provide a user-friendly and open-source implementation of available SSA algorithms accounting for delay effects, we sought out to develop the software package DelaySSA in three data-science languages popular in bioinformatics (R, Python and Matlab). Researchers familiar with any of these three languages could now perform SSA simulations conveniently. DelaySSA and tutorials with comprehensive examples and applications are available at https://github.com/Zoey-JIN/DelaySSA and https://github.com/FangZY-Lab/DelaySSA-dev.

## Design and implementation

**Fig 1:**
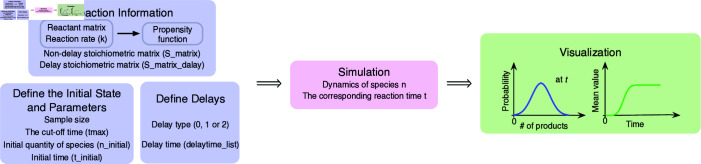
Design and implementation of DelaySSA. The input data for DelaySSA are reaction information, initial states, parameters and delays. Combining these into the simulation function, the temporal molecular states *n* at specific time *t* will be generated for further visualization.

In the stochastic simulation of biochemical reaction systems, Stochastic Simulation Algorithm (SSA)[1] is an important method that can describe the dynamic process of chemical reactions in the system with high accuracy. A detailed account of the theoretical background and algorithms could be found in S1 Appendix. Here, we implemented these algorithms with a common interface in the three most popular languages for systems biology, namely, R, Python, and Matlab (Fig A in S1 Appendix). Below we will illustrate the design considerations (Fig 1).

SSA can realistically reproduce the dynamic changes of species in the reaction system, including the intermediate processes of the system state. The core of the algorithm depends on the initial state of the system, including the types of species involved in the reaction and their initial quantities, as well as the reactions in the system. To prepare for simulation using the SSA, each reaction in the system must be described in detail, including reactants, products, and the rates of each reaction.

For non-Markovian processes with delays, we need to pay special attention to the characteristics of delayed reactions. The key to delayed reactions lies in two aspects, the changes in the quantities of reactants and products and the time points at which these changes occur. According to these criteria, reactions with delays can be divided into two categories: one with immediate changes in reactants at the start of the delay, and the other with latent changes in reactants at the end of the delay[21] (see S1 Appendix and Fig B in S1 Appendix for more comparison). For a reaction of the first type, the amount of reactants changes immediately at the beginning of the delay and generates products at the end of the delay when the reactions finish. Thus the amount of species is adjusted twice, both before and after the delay time *τ*. For a reaction of the other type, the amount of reactants does not change at the beginning of the delay when the reaction initiates, and it is not updated until the end of the delay. Thus the amount of reactants and of products are both updated at the end of the delay time *τ*. These two types of delayed reactions bring fundamental differences and diverge from the Markovian process which assumes an immediate change in response without any time delay.

In our implementation, reactant matrix represents the quantity of reactants (row of species and column of reactions). Reaction rate is the speed of chemical reaction. Two stoichiometric matrices are used to describe the non-delayed (S_matrix) and delayed (S_matrix_delay) reactions respectively. S_matrix represents the changes in molecular numbers for non-delayed reactions at the specific moment of reaction occurring. Each row corresponds to a species, while each column corresponds to a reaction. Each element of the matrix indicates the net changes of each species during each reaction. The quantity of species, the reaction rates, and the random numbers generated in the algorithm together specify when and which reaction is carried out. S_matrix_delay is used to describe changes in material quantities for reactions that involve delays. Similar to the S_matrix, the rows represent species, the columns represent reactions, and the elements indicate the net change in each species after the delay time *τ* has elapsed.

The delay time *τ* plays a critical role in the dynamics of delayed reactions. It can be either a fixed value or generated by a function, enabling more complex and realistic modeling of delays based on reactions.

The propensity function $f_{r}$ is fundamental for modeling reaction dynamics. We use the form of mass-action kinetics type [5] and $f_{r}(n)dt$ represents the probability of each reaction *r* occurring in the time interval *dt*, by taking into account the stochastic nature of the reaction process and the trend of the quantity of species. In combination with the random number generator, it determines which reaction to occur at each next moment and advances the simulation in discrete time steps. The system state is updated step by step iteratively until reaching a specific termination condition, either a maximum simulation time or a stable system state.

It is worth noting that each simulation results in one sequence or sample of the stochastic system. It is typically necessary to perform multiple repeated simulations to determine the average behavior of the system.

During the simulation, non-delayed reactions can influence the state of delayed reactions. For instance, if a non-delayed reaction causes a change in the quantity of a species that conflicts with the expected change from an ongoing delayed reaction, which must be resolved. A common method to handle such conflicts is to randomly remove one of the conflicting events from the delayed reactions. This approach maintains the consistency of the system state by preventing disruptions to the overall system dynamics caused by inconsistent species quantities.

## Results

### Evaluation on the Bursty and refractory models

To evaluate the validity of DelaySSA, we first applied it to two classical time-delayed models, the Bursty model and the refractory model.

**Fig 2:**
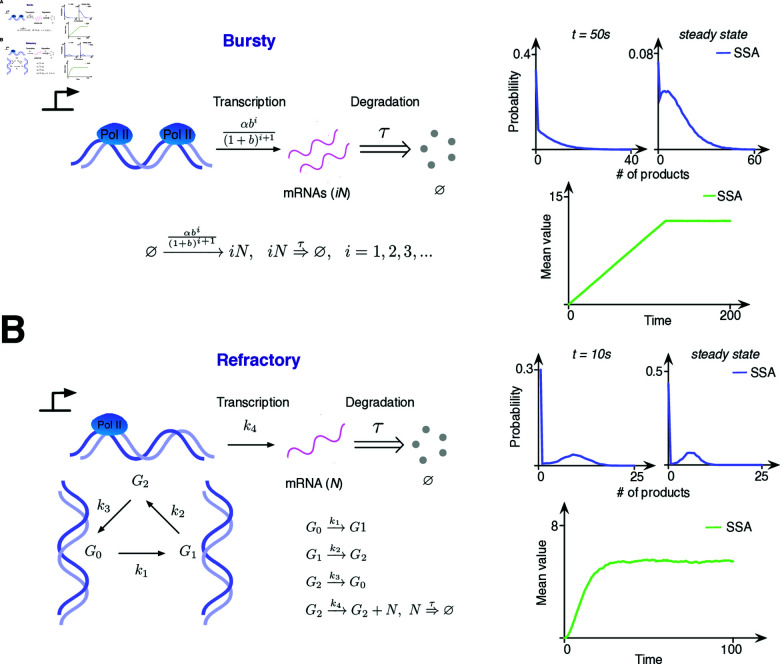
Bursty model and Refractory model simulation results. (A) Bursty model simulation results ($10^{5}$ samples). The production of nascent mRNA occurs in bursts and degrade after delay time *τ*. The burst frequency α=0.0282, the mean burst size b=3.46 and τ=120. (B) Refractory model simulation results ($10^{4}$ samples). The gene has three states, $G_{0}$, $G_{1}$ and $G_{2}$, and can switch from one state to another. Moreover, nascent mRNA is produced only when the gene is in state $G_{2}$. The parameters are $k_{1}=0.15$, $k_{2}=0.1$, $k_{3}=0.05$, $k_{4}=10$ and *τ* = 1.

The Bursty gene expression model describes the transcription bursts of mRNA [18,26] (Fig 2A). During short periods, multiple mRNA molecules are synthesized rapidly and then degraded after the time delay *τ*. Simulation results were consistent with the transcription rate as a function of burst frequency and burst size, and highlighted the phenomena that gene expression occurs in sporadic transcriptional bursts.

In the Refractory model [18,27,28], the gene state can switch between $G_{0}$, $G_{1}$ and $G_{2}$, but gene expression only occurs at $G_{2}$ (Fig 2B). The degradation of mRNA occurs after time delay *τ*. Simulation showed that mRNA expression has a bimodal distribution with a high probability of being zero, corresponding to refractory periods of remaining silence before switched to gene transcription, consistent with previous studies.

### Evaluation on the RNA velocity model

**Fig 3:**
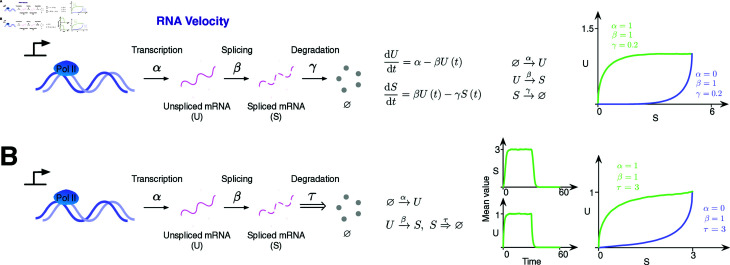
RNA Velocity model simulation results. (A) Simulation results for RNA Velocity model without delay ($2\;\times \;10^{4}$ samples). The simulation is composed of an up-regulation phase (both production and degradation) and a down-regulation phase (degradation-only). In the first phase, *%*, *%* and *%* for the reaction time t=50. In the second phase,  ∕ 1, *X* ∼ NB ( *r* , *μ* )  and *μ*. (B) Simulation results for RNA Velocity model with delay in degradation ($2\times 10^{4}$ samples). RNA degradation is specified as a delayed reaction. In the first (up-regulation) phase: *Y* ≡ *n*, *t* = 0 and *%*. In the second (down-regulation) phase: *%*, *%* and *%.*

RNA Velocity is a widely used model in computational methods inferring cell trajectories based on single-cell RNA-sequencing (scRNA-seq) data [7]. In this model, the unspliced mRNA (U) is initially produced at a rate denoted by *α*. The unspliced mRNA undergoes splicing at a rate *β* to generate mature spliced mRNA (S). After splicing, the mature mRNA undergo degradation at a rate *γ*. Simulation shows that before *α* becomes 0, the average values of U and S continue increasing until the steady state. In the down-regulation phase, *%* becomes 0, and the average value of U and S gradually decrease until returning to 0. The relationship between U and S can be seen from the U-S phase portrait (Fig 3A). This characteristic phase portrait is guaranteed by mRNA processing mechanism and have been widely accepted in single-cell data analysis to infer RNA-based cell differentiation. Since the gene transcriptional process (e.g. mRNA degradation) is inherent to time delays, and it still adheres to the maturation mechanism of mRNA, it would be reasonable to expect a similar U-S phase portrait after the introduction of time-delayed degradation. Indeed, when simulated with a time delay *%* in the mRNA degradation step, we again observed a similar U-S phase portrait (Fig 3B). Thus this simulation provides a further verification for DelaySSA.

**Fig 4:**
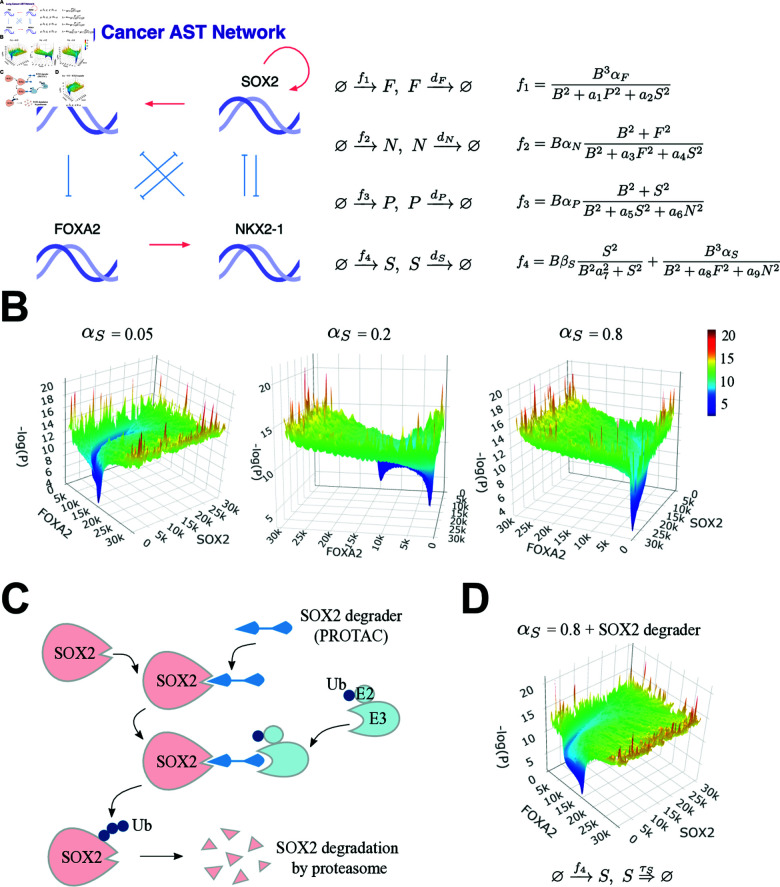
Modeling lung cancer AST network and its perturbation with a SOX2 degrader. (A) Lung Cancer AST Network and the corresponding stochastic equations. Parameters are $\alpha_{N}=\alpha_{P}=1$, $d_{F}=d_{N}=d_{P}=1$, $\alpha_{1}=\alpha_{2}=\alpha_{6}=\alpha_{8}=\alpha_{9}=1$, $\alpha_{F}=2.9$, $\beta_{S}=1.3$, $\alpha_{3}=0.8$, $\alpha_{4}=\alpha_{7}=2$, $\alpha_{5}=0.8$, $d_{S}=0.4$ with different $\alpha_{S}$. The control parameter is $\alpha_{S}$. (B) The potential landscape is approximated by simulating a large number of molecules corresponding to concentrations ($3\;\times \;10^{3}$ samples) with parameter values $\alpha_{S}=0.05$, $\alpha_{S}=0.2$, and $\alpha_{S}=0.8$, respectively representing the adenocarcinoma state, the intermediate state, and the squamous carcinoma state. FOXA2 and SOX2 amounts are chosen to visualize the landscape. (C) Principle of SOX2-targeted degradation by the PROTAC technique. The PROTAC molecule here is designed to recognize SOX2 proteins and to recruit E3 ligases to induce an addition of ubiquitin (Ub) proteins onto SOX2, which causes degradation of SOX2 subsequently. (D) Application of a SOX2 degrader to the squamous carcinoma state ($\alpha_{S}=0.8$) reprogram the AST network back to the adenocarcinoma state. Here the treatment effect of the SOX2 degrader is modeled as a delayed reaction ($\tau_{S}=1$).

### Application to concentration-based gene regulatory network models

We further consider applying DelaySSA to gene regulatory network models. One thing to note here is that, a number of such networks are described in concentration-based mathematical models, whereas SSA simulations are molecule-based. To better approximate concentration-based gene regulatory models, it would be useful to first perform a conversion from concentration-based models to molecule-based models. Here we demonstrate such a conversion in a lung cancer transition gene network model.

The network underlying lung cancer phenotypic transdifferentiation has been proposed and validated in recent studies of AST, which are closely related to drug-resistance during targeted therapies [10–12] (Fig 4A). The AST network has four core transcription factors: FOXA2 (*F*), NKX2-1 (*N*), P63 (*P*) and SOX2 (*S*). Among them, FOXA2 and NKX2-1 are in the adenocarcinoma lineage, whereas P63 and SOX2 are in the squamous carcinoma lineage.

The set of ODEs describing this network is as following [11]:


$$\{\begin{array}{c} \frac{dF}{dt}=\frac{\alpha_{F}}{1+a_{1}P^{2}+a_{2}S^{2}}-d_{F}F\\ \frac{dN}{dt}=\alpha_{N}\frac{1+F^{2}}{1+a_{3}F^{2}+a_{4}S^{2}}-d_{N}N\\ \frac{dP}{dt}=\alpha_{P}\frac{1+S^{2}}{1+a_{5}S^{2}+a_{6}N^{2}}-d_{P}P\\ \frac{dS}{dt}=\beta_{S}\frac{S^{2}}{a_{7}^{2}+S^{2}}+\frac{\alpha_{S}}{1+a_{8}F^{2}+a_{9}N^{2}}-d_{S}S \end{array}$$

All these proteins are described in terms of concentrations [11], and thus it is a prerequisite to convert to a molecule-based model by rewriting these ODEs.

Given the concentration (*x* moles per liter, or mol/L) and the number of molecules (*X*), we have the the relationship $X=xVN_{A}$, where $N_{A}\approx 6.023\times 10^{23}$ is the Avogadro constant denoting the number of molecules in one mole, and *V* is the system volume. Whenever the system volume is not specifically modeled, we can define $B=VN_{A}$ and simplify the relationship as *%*. In the following simulations, we used B=6023 that corresponds to a system volume of $V=10^{-20}$ liters [29].

Taking the first equation of the above AST network model as an example, we will show how to deduce its molecule-based version below. In this equation, FOXA2 is inhibited by P63 and SOX2 (the first term on the right-hand side) and undergoes spontaneous degradation (the second term),


$$\begin{array}{ccc} \frac{dF}{dt}=\frac{\alpha_{F}}{1+a_{1}P^{2}+a_{2}S^{2}}-d_{F}F & & \end{array}$$ (1)

Multiplying B to both sides of Eq. 1, we have


$$\frac{dF}{dt}B=B\frac{\alpha_{F}}{1+a_{1}P^{2}+a_{2}S^{2}}-Bd_{F}F,$$

$$\frac{dF}{dt}B=B\frac{B^{2}\alpha_{F}}{B^{2}(1+a_{1}P^{2}+a_{2}S^{2})}-Bd_{F}F,$$

$$\frac{d(FB)}{dt}=\frac{B^{3}\alpha_{F}}{B^{2}+a_{1}{\left(PB\right)}^{2}+a_{2}{\left(SB\right)}^{2}}-d_{F}(FB).$$

Here, *FB*, *PB*, and *SB* respectively represent the numbers of molecules corresponding to the concentrations *F*, *P*, and *S*. Thus, scaling the four molecules by a factor of *B*, we obtain the following molecule-based version of equation,


$$\begin{array}{ccc} \frac{dF}{dt}=\frac{B^{3}\alpha_{F}}{B^{2}+a_{1}P^{2}+a_{2}S^{2}}-d_{F}F. & & \end{array}$$ (2)

Similarly, We can obtain the other three equations. Together, the four molecule-based version of equations of the system are as following:


$$\{\begin{array}{c} \frac{dF}{dt}=\frac{B^{3}\alpha_{F}}{B^{2}+a_{1}P^{2}+a_{2}S^{2}}-d_{F}F\\ \frac{dN}{dt}=B\alpha_{N}\frac{B^{2}+F^{2}}{B^{2}+a_{3}F^{2}+a_{4}S^{2}}-d_{N}N\\ \frac{dP}{dt}=B\alpha_{P}\frac{B^{2}+S^{2}}{B^{2}+a_{5}S^{2}+a_{6}N^{2}}-d_{P}P\\ \frac{dS}{dt}=B\beta_{S}\frac{S^{2}}{B^{2}a_{7}^{2}+S^{2}}+\frac{B^{3}\alpha_{S}}{B^{2}+a_{8}F^{2}+a_{9}N^{2}}-d_{S}S \end{array}$$

### Modeling the lung cancer cell transition network with therapeutic interventions

We stochastically simulated the molecule-based equations to illustrate how the system state transits from the adenocarcinoma lineage to the squamous carcinoma lineage (Fig 4A). We sought out to portray the quasi-potential landscape of the network, which is also an evaluation of the capability of our package in simulating Waddington’s landscapes.

Stochastic simulation produces large numbers of molecules for each protein that better correspond to concentrations. We collected dynamical samples with random initiation and appropriate noise, estimated the frequency *P* in two-dimensional bins (FOXA2 and SOX2 as variables), and the quasi-potential *U* can be approximated through *%* (Fig 4B). When $\alpha_{S}$ is low (0.05), the system converges to a single stable point of FOXA2-high and SOX2-low, which corresponds to the lung adenocarcinoma phenotype (Fig 4B). When $\alpha_{S}$ is at an intermediate level (0.2), the system has two stable points simultaneously, suggesting a mixture of lung adenocarcinoma and squamous carcinoma phenotypes (Fig 4B). When $\alpha_{S}$ is large (0.8), the system again converges to a single stable point but of FOXA2-low and SOX2-high, which corresponds to the lung squamous carcinoma phenotype (Fig 4B). Taken together, stochastic simulation of this system clearly demonstrates the bistability of two phenotypic states of lung cancer, as well as the qualitative transition from FOXA2-high to SOX2-high when $\alpha_{S}$ increases dramatically.

From the therapeutic perspective, blocking lung cancer AST might reprogram squamous-state cells back to the original adenocarcinoma state and re-sensitize them to anticancer drugs. We can see that SOX2 plays a critical role in the AST network, based on above simulations, making it a seemingly candidate target for blocking AST. The technical challenge, however, is that transcription factors (TFs) like SOX2 are often considered “undruggable” with conventional inhibitors [30]. Recent advances in the proteolysis-targeting chimera (PROTAC) technology might alleviate this issue with rationally designed TF degraders [31]. In principle, SOX2-targeting PROTACs could be developed to degrade SOX2 (Fig 4C). Although research in this direction is still in its early stage [30] and it remains largely unclear whether SOX2 PROTACs would effectively block AST, we could test this rationale through model-based stochastic simulations. To model the effect of SOX2 degraders, we represent it as a delayed SOX2 protein degradation reaction (Fig 4D). Interestingly, whereas the system was stabilized in the squamous state given a large $\alpha_{S}$ (0.8), in silico addition of SOX2 degraders successfully produces a ‘reprogramming’ effect that pushes the system back to its original adenocarcinoma state (Fig 4D).

In conclusion, DelaySSA not only captures the potential landscapes underlying gene regulatory networks, but also proves to be highly valuable in simulating therapeutic interventions of network nodes.

### Computational efficiency

We have taken advantages of the Rcpp package to accelerate the simulation processes. Rcpp allows the incorporation of fast C++ routines within the R framework. In addition, we also employed a dynamical memory allocation mechanism to reduce the need of frequent memory allocation at each iteration during simulation, which reveals significant improvements for prolonged sampling.

We first compared the three delay algorithms in Rcpp-accelerated DelaySSA on the classical Bursty and Refractory models. DelayDirect and DelayRejection are faster on the Bursty model, whereas DelayMNR is faster on the Refractory model (Fig C in S1 Appendix). We further evaluated the Genetic Toggle Switch model with or without delays (see S1 Appendix for model details). There are slight differences among the three non-delay algorithms, whereas all three delay algorithms perform similarly (Fig D in S1 Appendix).

To compare the Rcpp-accelerated version of algorithms with the non-accelerated pure R version, we benchmarked the running time by simulation on all the three above models. As expected, optimization with Rcpp has significantly improved the computational efficiency for all algorithms, almost 10 times faster than their corresponding unoptimized versions on the Refractory and Toggle Switch models (Fig E in S1 Appendix). In comparison with the respective algorithms developed in Julia, a programming language featured by efficiency, Rcpp-optimized version of algorithms show a closely comparable performance (Fig E in S1 Appendix), indicating a high efficiency of the current DelaySSA implementation.

## Availability and future directions

DelaySSA (https://github.com/Zoey-JIN/DelaySSA and https://github.com/FangZY-Lab/DelaySSA-dev) currently implements a range of stochastic simulation algorithms with or without delays. For both simple and more complex biochemical gene networks, it already reaches a fast execution time. For extremely large-scale networks, further reducing the computation time remains a valuable direction in the future. Interesting explorations include integration of multi-scale approaches, hybrid deterministic-stochastic algorithms, and dimensionality-reduction methods.

In biological research, stochastic simulations offer several advantages compared to actual biological experiments. Experiments typically require significant expenditure whereas simulations primarily depend on computational resources. Biomedical experiments are often time-consuming whereas simulations can be completed in a shorter time. In certain cases, experimental techniques might not even yet be fully developed (such as in the above SOX2-targeting example). In all these scenarios, stochastic simulations offer a unique and important value in rationale development or evaluation.

Given the importance of SSA in simulation of biochemical and molecular networks, it is surprising that few user-friendly packages have been developed in popular programming languages such as R, Python and Matlab, especially those non-Markovian algorithms capable of handling time delays inherent in more realistic biological systems. This new implementation DelaySSA fills the current gap in three programming languages altogether, thus providing a powerful platform for enormous researchers from different backgrounds and for applications in diverse fields. By integrating both traditional SSA and delayed SSA, DelaySSA provides a unified and intuitive interface for a wide range of stochastic simulations, allowing users to describe and simulate reaction networks with minimal coding effort. In addition, DelaySSA does not require additional dependencies, simplifying the installation and setup process. A series of concrete tutorials are also accompanied in the Github websites of DelaySSA.

One of the significant benefits of DelaySSA is its ability to generate accurate simulation data. This feature is particularly valuable for researchers who may lack precise experimental data [32–35], enabling them to test and improve biological models, and to optimize or fine tune computational methods according to simulated data. As we have demonstrated in the lung cancer AST intervention example, although targeting SOX2 with PROTAC is practical in principle, in reality it is not yet fully established. Nonetheless, with DelaySSA we could simulate a therapeutic intervention model with the SOX2-targeting PROTAC modeled as a delayed degradation reaction. Our simulation data allow a portrait of the ‘reprogrammed’ system state landscape after SOX2-degrader intervention, theoretically predicting the efficacy of SOX2-based intervention for blocking lung cancer AST. This provides a new valuable clue for future research on blocking drug-resistant AST in lung cancer [12,36,37]. Following our application case, researchers could further perform valuable applications to reveal novel biomedical insights in various studies of systems biology.

## Supporting information

S1 Appendix Mathematical foundations and supplementary data. The S1 Appendix file contains Mathematical Foundations, Algorithmic Details, and Supplementary Data which includes five supplementary figures.
(PDF)
