# Supplementary material for: DelaySSA: stochastic simulation of biochemical systems and gene regulatory networks with or without time delays
Source: PLoS Comput Biol. 2025 Apr 8;21(4):e1012919. doi: 10.1371/journal.pcbi.1012919 (PMC11977973; doi:10.1371/journal.pcbi.1012919)
Supplement: S1 Appendix — Mathematical foundations and supplementary data. The S1 Appendix file contains Mathematical Foundations, Algorithmic Details, and Supplementary Data which includes five supplementary figures. (PDF) [file pcbi.1012919.s001.pdf]

DelaySSA: stochastic simulation of biochemical systems and gene regulatory networks with or without time delays

Contents

1 Mathematical Foundations and Algorithmic Details **1**

1.1 Basic Concepts 1

1.2 Gillespie Algorithm 2

1.3 The Next Reaction Method Algorithm 3

1.4 Modified Next Reaction Method Algorithm 3

1.5 Delay Direct Method Algorithm 4

1.6 Delay Modified Next Reaction Method Algorithm 6

1.7 Delay Rejection Method Algorithm 6

2 Supplementary Data **6**

2.1 Choice of implementation languages 6

2.2 Two categories of time-delayed reactions 7

2.2.1 Example 1 7

2.2.2 Example 2 8

2.3 Computational efficiency of different stochastic simulation algorithms 8

2.3.1 Bursty model and Refractory model 8

2.3.2 Genetic toggle switch model 9

2.3.3 Efficiency of implementations 9

1 Mathematical Foundations and Algorithmic Details

Here we provide a detailed account of the mathematical foundations for SSA methodology and algorithms with or without delays.

1.1 Basic Concepts

By conceptualizing chemical and biological processes as a series of discrete chemical reactions, we can mathematically formalize them using the Stochastic Simulation Algorithm (SSA) framework. Several algorithms have been developed for classical SSA: Gillespie (Direct) [1], Next Reaction Method (NR) [2], and Modified Next Reaction Method (MNR) [3].

On the other hand, biochemical reactions (such as gene transcription and translation) may require certain temporal durations to complete after their initiation. As a result, time delays might be inherent to such processes, making classical SSA algorithms unsuitable for modeling them. To remedy this, several modified algorithms are designed to handle time delays: Delay Direct Method (DelayDirect) [4], Delay Rejection Method (DelayRejction) [5] and Delay Modified Next Reaction Method (DelayMNR) [3].

Here we first present the basic concepts of these SSA algorithms.

Given a finite set of chemical species $X_i, i = 1, \ldots, N$, and R chemical reactions, we define the reactions by the notation

$$\sum_{i=1}^{N} s_{ir} X_i \xrightarrow{k_r} \sum_{i=1}^{N} s_{ir}^{'} X_i, \quad r = 1, \ldots, R$$

where s_{ir} and s'_{ir} denote the numbers of reactant and product molecules, respectively, and k_r is the reaction rate constant of the $r-$th reacion. The stoichiometric matrix S is given by

$$S_{ir} = s^{'}_{ir} - s_{ir}, \quad r = 1, \dots, R, \quad i = 1, \dots, N$$

According to [6], propensity function $f(\mathbf{n})$ are in the form of mass-action kinetics type

$$f_r(\mathbf{n}) {=} k_r \Omega \prod_{i=1}^{N} \frac{n_i!}{(n_i - s_{ir})! \Omega^{s_{ir}}}$$

where $\mathbf{n} = (n_1, \dots, n_N)$, n_i is the number of species X_i, and Ω is the volume of the system.

Time delays could be either a constant or a stochastic value from some probability distribution. According to [5], reactions with delays are categorized into consuming and nonconsuming reactions. If a delayed reaction is a nonconsuming reaction, it initiates at t and will finish until $t + t_{\text{delay}}$, then the $\mathbf{n}$ of the number of species will only change at $t + t_{\text{delay}}$ (i.e. end). If a delayed reaction is a consuming reaction, and it initiates at t and will finish until $t + t_{\text{delay}}$, then the $\mathbf{n}$ of the number of species will change both at t (i.e. start) and $t + t_{\text{delay}}$ (i.e. end).

If taking non-delayed reaction together, then we can categorize reactions into the following three cases.

Case 1: If reaction r loses the reactant species and gains the product species at the initiation time t, we denote the reaction r without delays as ND.

Case 2: If reaction r loses the reactant species and gains the product species at the completion time $t + t_{\text{delay}}$, we denote the reaction r with delays as CD.

Case 3: If reaction r loses the reactant species and gains the product species respectively at the initiation time t and the completion time $t + t_{\text{delay}}$, we denote the reaction r with delays as ICD.

1.2 Gillespie Algorithm

Consider a system of N chemical species and R chemical reactions, each with a propensity function $f_r(n)$. It is assumed that the time to complete each reaction is negligible. During the stochastic simulation, it calculates the time for the next reaction (τ) and chooses the next reaction (μ). Another fundamental assumption underlying SSA methods [4] is:

$$f_r(n(t))dt = \text{the probability that reaction r occurs in a small time interval } [t, t + dt)$$

Following the assumption, τ and μ are two independent random variables with the probability density functions

$$p(\tau | n, t) = \lambda(n, t) \exp(-\tau \lambda(n, t)), \quad \lambda = \sum_{r=1}^{R} f_r(n, t)$$

$$p(\mu | n, t) = f_r(n, t) / \lambda(n, t)$$

Accordingly, τ and μ can be generated as

$$\tau = -\ln(u_1) / \lambda(n, t)$$

$$\mu = \text{the integer satisfies} \sum_{r=1}^{\mu-1} f_r(n, t) < u_2 \lambda(n, t) \leq \sum_{r=1}^{\mu} f_r(n, t)$$

where $u_1, u_2 \sim \text{Uniform}(0, 1)$ respectively.

The pseudocode of Gillespie Algorithm is shown in Algorithm 1.

Algorithm 1 Gillespie Algorithm

1: Initialize. Set $t = 0$ and species number $n = n_{\text{initial}}$.
2: Calculate the propensity function, f_r, for each reaction.
3: Generate two independent Uniform $(0, 1)$ random numbers, u_1 and u_2.
4: Set $\tau = -\ln(u_1)/\Sigma_{r=1}^{R} f_r$.
5: Find the integer μ which satisfies $\Sigma_{r=1}^{\mu-1} f_r < u_2 \Sigma_{r=1}^{R} f_r \leq \Sigma_{r=1}^{\mu} f_r$.
6: Set $t = t + \tau$.
7: Update species numbers n upon the completion of the reaction μ.
8: Return to step 2 or quit.

1.3 The Next Reaction Method Algorithm

Let v_r, $v'_r \in N \geq 0^N$ be the vectors representing the number of each species consumed and produced in the r-th reaction, respectively. Then, if $N_r(t)$ is the number of initiations of reaction r by time t, the state of the system at time t is

$$n(t) = n(0) + \sum_{r=1}^{R} N_r(t)(v'_r - v_r)$$

According to the fundamental assumption, the counting process $N_r(t)$ (with intensity $f_r(n(t))$) satisfies $p(N_k(t + \Delta t) - N_k(t) = 1) = f_r(n(t))\Delta t$, where Δt is small. Thus,

$$N_r(t) = Y_r\Big(\int_0^t f_r(n(s))ds\Big)$$

Here, Y_r are independent Poisson processes with unit rate. For each reaction r, let $T_r(t) = \int_0^t f_r(n(s))ds$, which can be understood as the internal time of Y_r.

Let $\Delta = \min_r \Delta t_r$, where Δt_r is the gap time for the r-th reaction. Let $\bar{t} = t + \Delta$, and $\overline{f_r}$ be the updated propensity function.

The internal time of the next occurrence of Y_r remains $T_r(t) + f_r\Delta t_r$. The updated internal time of Y_r is $T_r(\bar{t}) = T_r(t) + f_r\Delta$. As a result, the internal time before executing the r-th reaction is actually the difference:

$$(T_r(t) + f_r\Delta t_r) - (T_r(t) + f_r\Delta) = f_r(\Delta t_r - \Delta)$$

Thus, the absolute time that have to pass before executing the r-th reaction, $\Delta\bar{t}_r$, can be derived by solving $\overline{f_r}\Delta\bar{t}_r = f_r(\Delta t_r - \Delta)$. And we get

$$\overline{\tau_r} = f_r/\overline{f_r}(\Delta t_r - \Delta) + \bar{t} = f_r/\overline{f_r}((t + \Delta t_r) - (t + \Delta)) + \bar{t} = f_r/\overline{f_r}(\tau_r - \bar{t}) + \bar{t}$$

Thus the absolute times for each $r \neq \mu$ is obtained, without any additional random number generation.

Note that after the first timestep, all subsequent timesteps only require one random number generation. This algorithm adopted the notion of a dependency graph and a priority queue for computational efficiency [2], which is similar to the random number generation for each step of the original Gillespie's direct algorithm. The pseudocode of The Next Reaction Method is shown in Algorithm 2.

1.4 Modified Next Reaction Method Algorithm

According to [3], Modified Next Reaction Method Algorithm is equivalent to the Next Reaction Method Algorithm, except that making more explicit use of the internal time T_r. The key formula of this algorithm is $\Delta t_r = (1/f_r)(P_r - T_r)$. The pseudocode of Modified Next Reaction Method Algorithm is shown in pseudocode 3.

Algorithm 2 The Next Reaction Method Algorithm

1: Initialize. Set $t = 0$ and set species number $n = n_{\text{initial}}$.
2: Calculate the propensity function, f_r, for each reaction.
3: Generate R independent Uniform$(0, 1)$ random numbers, u_r.
4: set $\tau_r = -\ln(u_r)/f_r$.
5: Set $t = \min_r\{\tau_r\}$. Here we assume that τ_μ is the minimum.
6: Update species number n based upon the completion of the reaction μ.
7: Recalculate the propensity function, $\overline{f_r}$, for each reaction.
8: For each $r \neq \mu$, set $\tau_r = (f_r/\overline{f_r})(\tau_r - t) + t$.
9: For reaction μ, let u' be Uniform $(0, 1)$ and set $\tau_\mu = -\ln(u')/\overline{f_\mu} + t$. If τ_r is either NA or Inf, it also needs to be recalculated in this manner.
10: For each r, set $f_r = \overline{f_r}$.
11: Return to step 5 or quit.

Algorithm 3 Modified Next Reaction Method Algorithm

1: Initialize. Set $t = 0$ and set species number $n = n_{\text{initial}}$. For each $r \leq R$, set $P_r = 0$ and $T_r = 0$.
2: Calculate the propensity function, f_r, for each reaction.
3: Generate R independent Uniform $(0, 1)$ random numbers, u_r, and set $P_r = -\ln(u_r)$.
4: Set $\tau_r = (P_r - T_r)/f_r$.
5: Set $\tau = \min_r\{\tau_r\}$. Here we assume that τ_μ is the minimum.
6: Set $t = t + \tau$. And update species number n based upon the completion of the reaction μ.
7: For each r, set $T_r = T_r + f_r\tau$.
8: For reaction μ, let u' be Uniform $(0, 1)$ and set $P_\mu = P_\mu - \ln(u')$.
9: Recalculate the propensity function, f_r, for each reaction.
10: Return to step 4 or quit.

1.5 Delay Direct Method Algorithm

Assume N_d delayed reactions are initiated at the time t, and supposed to complete at $t + T_1, \ldots, t + T_{N_d}$, where $T_1 \leq T_2 \leq \ldots \leq T_{N_d}$. As in the derivation of Gillespie's exact algorithm, from the fundamental assumption, we have $p(\tau, \nu)d\tau = p_0(\tau)f_\mu(t+\tau)d\tau$, where $p_0(\tau)$ is the probability of no reactions taking place in $[t, t+\tau)$ and equals to $\exp(-\Sigma_{j=0}^{i-1}\lambda(t+T_j)(T_{j+1}-T_j) - \lambda(t+T_i)(\tau - T_i))$, $\tau \in [T_i, T_{i+1})$, $i = 0, \ldots, N_d$, where the exponent assumes to be zero when $i = 0$

$$p(\tau|\mathbf{n}, t) = \lambda(t + T_i)\exp(-\sum_{j=0}^{i-1}\lambda(t + T_j)(T_{j+1} - T_j) - \lambda(t + T_i)(\tau - T_i)), \quad \lambda(t + T_i) = \sum_{r=1}^{R} f_r(t + T_i)$$

$$p(\mu|\mathbf{n}, t) = f_r(t + T_i)/\lambda(t + T_i), \quad \mu = 1, \ldots, R, \quad \tau \in [T_i, T_{i+1}), \quad i = 0, \ldots, N_d$$

Consequently, generation of τ and μ follows from

$$\tau = T_i + \frac{-\ln(1 - u_1) - \Sigma_{j=0}^{i-1}\lambda(t + T_j)(T_{j+1} - T_j)}{\lambda(t + T_i)}$$

$$\mu = \text{the integer satisfies} \sum_{r=1}^{\mu-1} f_r(t + T_i) < u_2\lambda(t + T_i) \leq \sum_{r=1}^{\mu} f_r(t + T_i)$$

where $u_1, u_2 \sim$ Uniform$(0, 1)$.

The pseudocode of Delay Direct Method Algorithm is shown in Algorithm 4. Notice that in the pseudocode, we modified step 19 in the original algorithm [4] for computational efficiency while maintaining equivalence.

Algorithm 4 Delay Direct Method Algorithm

Require: Assume at time t there are initiated delayed reactions set to complete respectively at times $t+T_1, t+T_2, \ldots, t+T_d$. Define $T_0 = 0$ and $T_{d+1} = \infty$. Define $Tstruct$, whose i-th $(i = 1, \ldots, d)$ row stores T_i and the index μ_i to the corresponding reactions.

1: Initialize. Set $t = 0$ and set species number $n = n_{\text{initial}}$. Create a empty $Tstruct$.
2: Calculate propensity functions $f_r(t), r = 1, \ldots, R$.
3: Generate τ.
4: Generate an independent Uniform$(0, 1)$ random number u_1.
5: If $Tstruct$ is empty, there are no ongoing delayed reactions.
6: Set $\tau = -\ln(u_1)/\Sigma_{r=1}^{R} f_r$.
7: Else
8: Set $a_{\text{mask}} = 0$.
9: Set $i = 0$, $F = 0$ and $a_t = \Sigma_{r=1}^{R} f_r T_1$.
10: While $F < u_1$
11: Calculate $F = 1 - e^{-a_t}, i = i + 1$.
12: Calculate propensity $f_r(t + T_i)$ due to the completion of the delayed reaction at $t + T_i$, and update $\Sigma_{r=1}^{R} f_r(t + T_i)$.
13: Set $a_{mask} = a_t$. Update $a_t = a_t + \Sigma_{r=1}^{R} f_r(t + T_i)(T_{i+1} - T_i)$.
14: If $i > 1$, update species number n due to the delay reaction at $t + T_{i-1}$
15: End while
16: Set $i = i - 1$.
17: Calculate $\tau = T_i - (\ln(1 - u_1) + a_{\text{mask}} - \Sigma_{r=1}^{R} f_r(t + T_i)(T_{i+1} - T_i))/\Sigma_{r=1}^{R} f_r(t + T_i)$.
18: End if
19: If $\tau \in [T_i, T_{i+1})$, delete the columns $1, \ldots, i$ of T_i and set $T_j = T_j - \tau$.
20: Generate u_2 from a Uniform(0,1) random variable, and find the integer μ which satisfies $\Sigma_{r=1}^{\mu-1} f_r < u_2 \Sigma_{r=1}^{R} f_r \leq \Sigma_{r=1}^{\mu} f_r$.
21: Update according to the type of reaction μ: if the reaction μ belongs to type ND, update species number $\mathbf{n}$; if the reaction belongs to type CD, store the time $t + \tau_\mu$; if the reaction belongs to type ICD, update species number $\mathbf{n}$ and store the time $t + \tau_\mu$. If it is a delayed reaction (CD or ICD), insert τ_μ and the reaction μ into $Tstruct$, ensuring that the times in $Tstruct$ remain in ascending order.
22: Set $t = t + \tau$.
23: Return to step 2 or quit.

1.6 Delay Modified Next Reaction Method Algorithm

Delay Modified Next Reaction Method Algorithm is modified Next Reaction Method for systems with delays. According to [3], let T_r be the current internal time of Y_r, P_r be the first internal time after T_r when Y_r completes, and f_r be the propensity function for the $r-$th reaction, then the time until the next initiation of reaction r remains $\Delta t_r = (1/f_r)(P_r - T_r)$ (assuming no initiation or completion of other reactions). Assign a vector, s_r, to each delayed reaction, which stores the completion times of the $r-$th reaction in ascending order. We then know that the next change (either initiation or completion) of the system state will be at the time

$$\Delta = \min\{\Delta t_r, s_r[1] - t\}$$

from the current time t. The pseudocode of Delay Modified Next Reaction Method Algorithm is shown in Algorithm 5.

Algorithm 5 Delay Modified Next Reaction Method Algorithm

```
1:  Initialize. Set t = 0 and set species number n = n_initial. For each r ≤ R, set P_r = 0, T_r = 0, and
    s_r = [∞].
2:  Calculate for each reaction the propensity function f_r.
3:  Generate R independent Uniform(0,1) random numbers, u_r, and set P_r = −ln(u_r).
4:  Set τ_r = (P_r − T_r)/f_r.
5:  Set τ = min_r{τ_r, s_r[1] − t}.
6:  Set t = t + τ.
7:  If the delayed reaction μ completes:
8:     Update species number n based upon the completion of the reaction μ.
9:     Delete the first element of S_μ.
10: Elseif reaction μ initiated and μ ∈ ND
11:    Update species number n according to reaction μ.
12: Elseif reaction μ initiated and μ ∈ CD
13:    Update s_μ by inserting t + τ_μ into s_μ ensuring that the times in s_μ remain in ascending order.
14: Elseif reaction μ initiated and μ ∈ ICD
15:    Update species number n based upon the initiation of reaction μ.
16:    Update s_μ by inserting t + τ_μ into s_μ ensuring that the times in s_μ remain in ascending order.
17: For each r, set T_r = T_r + f_r τ.
18: If reaction μ initiated, let u' be Uniform(0,1) and set P_μ = P_μ − ln(u').
19: Recalculate the propensity functions, f_r.
20: Return to step 4 or quit.
```

1.7 Delay Rejection Method Algorithm

Simulation methods for systems with delays have to schedule when such delayed reactions initiate and complete. The propensity functions can change at initiation times. The Rejection Method for delay systems is proposed in [5]. This algorithm computes the initiation times exactly like the original Gillespie Algorithm except that when a stored delayed reaction completes, the corresponding timestep is discarded, and the system state is updated to incorporate that delayed reaction. It then attempts for another timestep initiated at its new state. The pseudocode of Delay Rejection Method Algorithm is shown in Algorithm 6.

2 Supplementary Data

2.1 Choice of implementation languages

Since our motivation is to provide a unified suite of SSA algorithms for systems biology, we evaluated those languages frequently chosen for open-source projects related to systems biology. As we can see, the

Algorithm 6 Delay Rejection Method Algorithm

1: Initialize. Set $t = 0$ and set species number $n = n_{\text{initial}}$.
2: Calculate propensity functions $f_r(t), r = 1, \ldots, R$.
3: Generate u_1 from a Uniform(0,1) random variable, and set $\tau = -\ln(u_1)/\Sigma_{r=1}^{R} f_r$.
4: If there is a delayed reaction to finish in $[t, t+\tau)$
5: Discard τ.
6: Update t to be the time of the first next delayed reaction and update the species number.
7: Return to step 2 or quit.
8: Else if there is no delayed reaction in $[t, t+\tau)$.
9: Generate u_2 from a Uniform(0,1) random variable, and find the integer μ which satisfies $\Sigma_{r=1}^{\mu-1} f_r < u_2 \Sigma_{r=1}^{R} f_r \leq \Sigma_{r=1}^{\mu} f_r$.
10: Update according to the type of reaction μ belongs to: if the reaction μ belongs to type ND, update species number $\mathbf{n}$; if the reaction belongs to type CD, store the time $t + \tau_\mu$; if the reaction belongs to type ICD, update species number $\mathbf{n}$ and store the time $t + \tau_\mu$.
11: Set the time $t = t + \tau$.
12: Return to step 2 or quit.

three languages implementing DelaySSA together occupy a large proportion in those projects (Fig A).

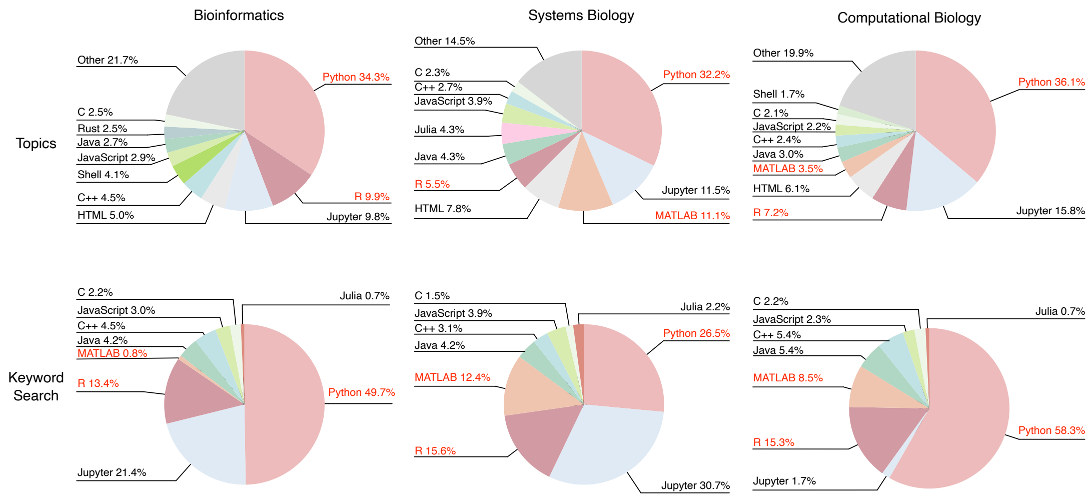

Fig A. The proportion of programming languages used in GitHub under selected topics or keywords. R, Python, and MATLAB are highlighted.

2.2 Two categories of time-delayed reactions

In order to better illustrate the differences between the two types of delayed reactions, we construct two synthetic examples (see Fig B) and compare the temporal dynamics of the average amounts of species under the two delay configurations.

2.2.1 Example 1

This example represents a reaction system where A is a functional non-coding RNA, B and C are coding RNAs transcribed in different ways (either with or without delays) to produce the protein P. Moreover, B is generated under the influence of A. Now this system can be expressed as

$$\begin{aligned} &A \xrightarrow{k_1} A + B, \quad A + B \overset{\tau}{\Rightarrow} A + P, \\ &\varnothing \xrightarrow{k_2} C, \quad A + C \xrightarrow{k_3} A + P \end{aligned} \tag{S1}$$

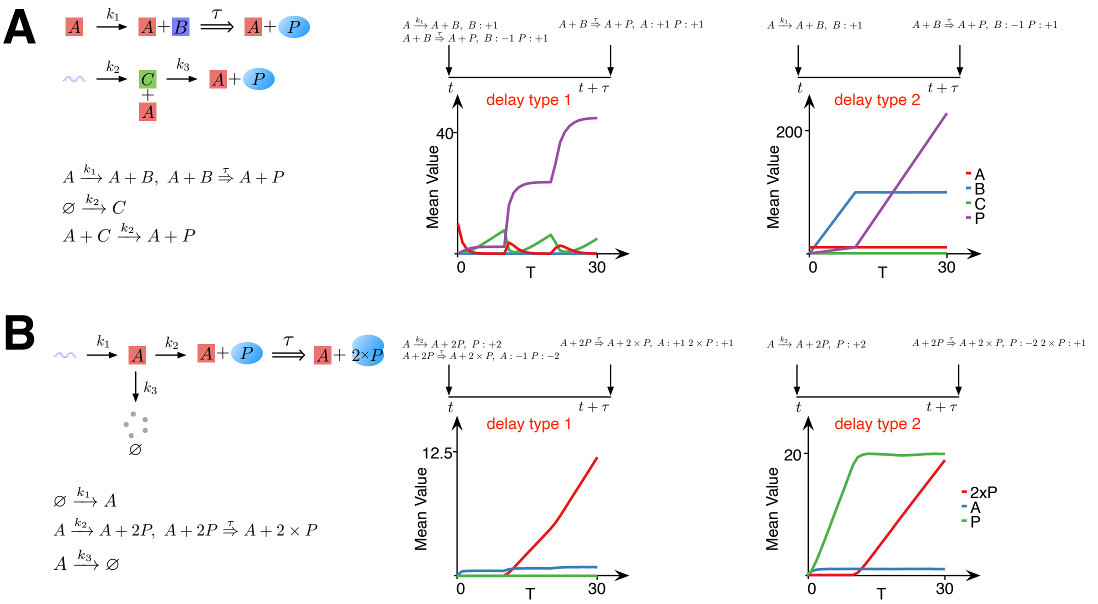

Fig B. Stochastic simulation results of the two models under two different delay types. A. Stochastic simulation results for the two delay types of Example 1. The rate parameters used are $k_1 = 1$, $k_2 = 1$, $k_3 = 1$, $\tau = 10$ with $C = 10$ as the initial value. B. Stochastic simulation results for the two delay types of Example 2. The rate parameters used are $k_1 = 1$, $k_2 = 1$, $k_3 = 1$, $\tau = 10$.

In the first type of delay, we assume that at time t, after A and B are generated, they immediately disappear at time t, and after a certain delay at time $t + \tau$, A and P are generated. In the second type of delay, we assume that at time t, after A and B are generated, they remain in the system for reactions and disappear only at time $t + \tau$, producing A and P (panel A of Fig B). Under the initial conditions with a certain amount of C, the temporal behaviours of these delay types show significant differences.

2.2.2 Example 2

In the example 2, A promotes both the generation of P and the dimerization of P, which can be expressed as

$$\varnothing \xrightarrow{k_1} A, \quad A \xrightarrow{k_2} A + 2P, \quad A + 2P \overset{\tau}{\Rightarrow} A + 2 \times P, \quad A \xrightarrow{k_3} \varnothing \tag{S2}$$

In the first type of delay, we assume that at time t, a reaction to generate P occurs. At time t, A and two molecules of P immediately disappear, and after a certain delay, at time $t + \tau$, A and the dimer of P are generated. In the second type of delay, we assume that at time t, a reaction to generate P occurs. A remains in the system and can participate in other reactions until at time $t + \tau$, when A and two P molecules disappear, leading to the generation of the dimer protein. The stoichiometric equations describe the generation and consumption of each species. In the first type of delay, since P is generated and immediately disappears, there is no accumulation of the species, and after the delay, it accumulates rapidly, showing a clear post-delay growth (panel B of Fig B). In the second type of delay, there is an accumulation process for P until a turning point occurs when the delayed reaction takes place.

The two examples above highlight the different impacts of the two types of delays on system dynamics, revealing the complexity introduced by delay mechanisms in complex reaction networks.

2.3 Computational efficiency of different stochastic simulation algorithms

2.3.1 Bursty model and Refractory model

We tested the computational time for the Bursty model and the Refractory model under different stochastic simulation algorithms (Fig C). Apple M2 Pro was used for timing in this experiment.

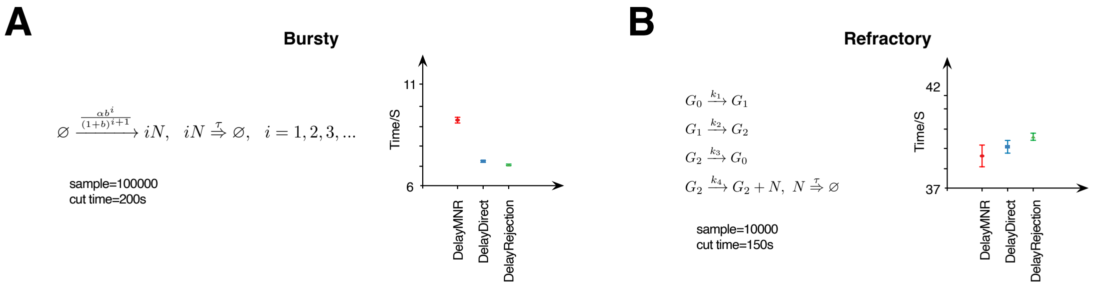

Fig C. Benchmark of delay algorithms on the Bursty model and the Refractory model. Mean and standard deviation of the running time under different stochastic simulation algorithms in DelaySSA (50 independent replicates). Here "sample" represents the number of SSA iterations, and "cut time" represents the maximum reaction time in each sample.

2.3.2 Genetic toggle switch model

We further benchmarked on the genetic toggle switch model (panel A of Fig D), which can be expressed as [7–10]

$$\begin{aligned}
&G_x \xrightarrow{\rho_x} G_x + P_x, \quad G_y \xrightarrow{\rho_y} G_y + P_y,\\
&2P_x + G_y \xrightarrow{\sigma_x} \hat{G}_y, \quad 2P_y + G_x \xrightarrow{\sigma_y} \hat{G}_x,\\
&\hat{G}_y \xrightarrow{\hat{\sigma}_x} 2 \times P_x + G_y, \quad \hat{G}_x \xrightarrow{\hat{\sigma}_y} 2 \times P_y + G_x,\\
&P_x \xrightarrow{\beta_x} \varnothing, \quad P_y \xrightarrow{\beta_y} \varnothing
\end{aligned} \tag{S3}$$

$\hat{G}_x$ and $\hat{G}_y$ represent genes inactivated by suppressive protein binding. The total copy numbers of each gene are constrained by $G_x + \hat{G}_x = 1$ and $G_y + \hat{G}_y = 1$. P_x and P_y represent the monomer forms of each protein, with synthesis rates of ρ_x and ρ_y, respectively, and degradation rates of β_x and β_y, respectively. $2 \times P_x$ and $2 \times P_y$ represent dimer forms of each protein, with dimerization rates of κ_x and κ_y, respectively. The gene-binding rates for protein dimers are respectively σ_x and σ_y, and their dissociation rates are respectively $\hat{\sigma_x}$ and $\hat{\sigma_y}$. The rate parameters used in this case study are $\rho_x = 50$, $\rho_y = 50$, $\beta_x = 1$, $\beta_y = 1$, $\sigma_x = 10^{-4}$, $\sigma_y = 10^{-4}$, $\hat{\sigma}_x = 0.1$, $\hat{\sigma}_y = 0.1$.

Then we extended the genetic toggle switch model to the delay situation (panel B of Fig D). We consider that P_x and P_y disappear after a fixed time τ after they are generated, instead of the form of first-order degradation. The genetic toggle switch model with time delay can be expressed as

$$\begin{aligned}
&G_x \xrightarrow{\rho_x} G_x + P_x, \quad G_y \xrightarrow{\rho_y} G_y + P_y,\\
&2P_x + G_y \xrightarrow{\sigma_x} \hat{G}_y, \quad 2P_y + G_x \xrightarrow{\sigma_y} \hat{G}_x,\\
&\hat{G}_y \xrightarrow{\hat{\sigma}_x} 2 \times P_x + G_y, \quad \hat{G}_x \xrightarrow{\hat{\sigma}_y} 2 \times P_y + G_x,\\
&P_x \overset{\tau_x}{\Rightarrow} \varnothing, \quad P_y \overset{\tau_y}{\Rightarrow} \varnothing
\end{aligned} \tag{S4}$$

The rate parameters used in this case study are $\rho_x = 50$, $\rho_y = 50$, $\tau_x = 10$, $\tau_y = 10$, $\sigma_x = 10^{-4}$, $\sigma_y = 10^{-4}$, $\hat{\sigma}_x = 0.1$, $\hat{\sigma}_y = 0.1$. We used the Apple M2 Pro for timing in our experiment.

2.3.3 Efficiency of implementations

We benchmarked three implementations (Rcpp-R, Pure-R, and Julia) in all the three above models: Bursty, Refractory, and Toggle Switch (Fig E). Although Pure-R implementation is much slower, the Rcpp-optimized implementation is significantly accelerated, at a high efficiency comparable to that of the Julia implementation.

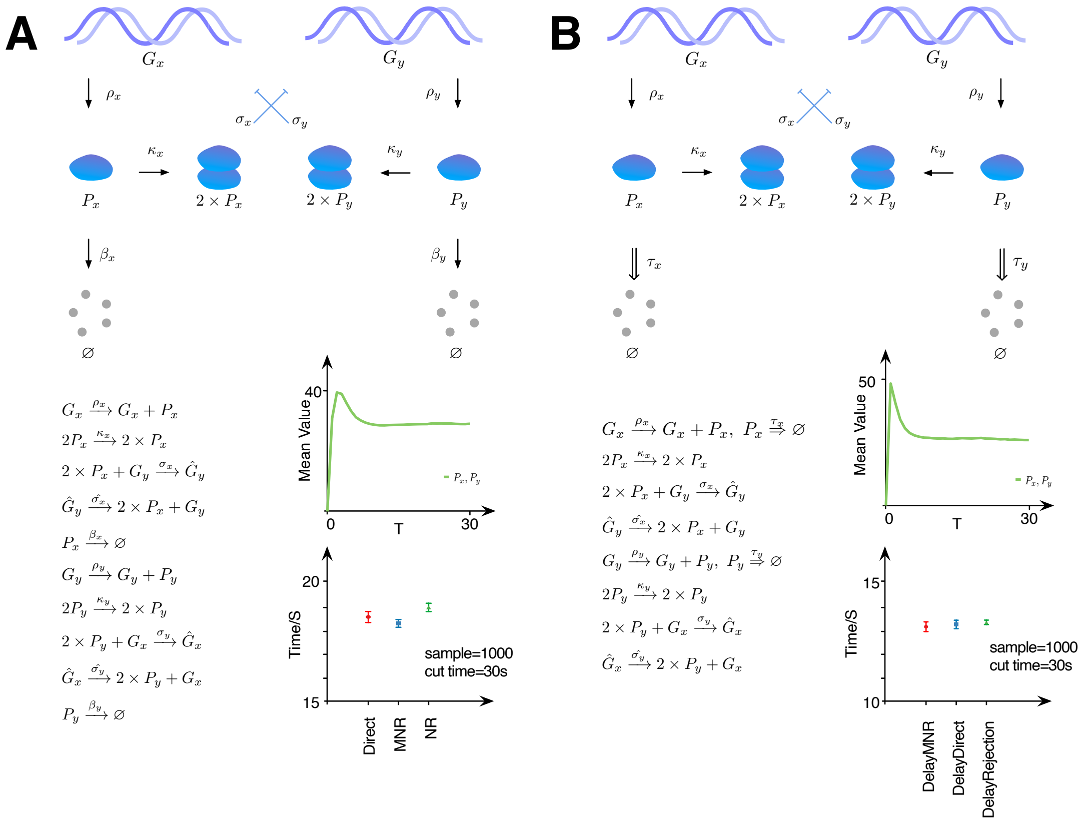

Fig D. Simulation of the genetic toggle switch model with and without time delays. The P_x and P_y curves are overlapping. "sample": number of replicates; "cut time": maximum reaction time.

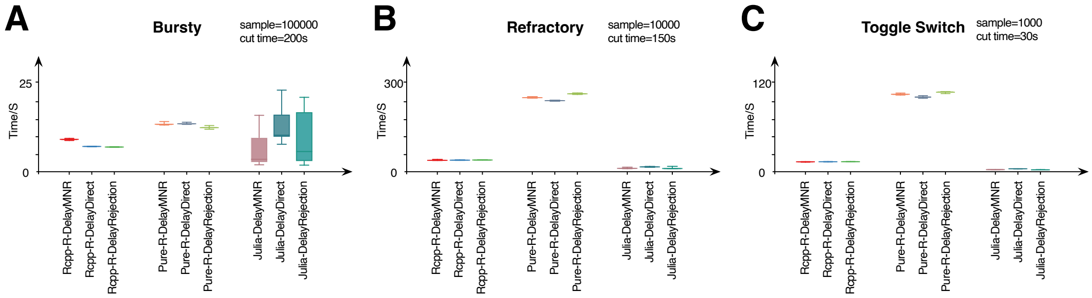

Fig E. Benchmark on three implementations. Three implementations are evaluated: Rcpp-optimized R-DelaySSA, Pure R-DelaySSA, and Julia implementation. Parameters are the same as above. The parameters of Bursty model are $\alpha = 0.0282$, $b = 3.46$. The parameters of Refractory model are $k_1 = 0.15$, $k_2 = 0.1$, $k_3 = 0.05$, $k_4 = 10$ and $\tau = 1$. The rate parameters used in Toggle Switch model are $\rho_x = 50$, $\rho_y = 50$, $\beta_x = 1$, $\beta_y = 1$, $\sigma_x = 10^{-4}$, $\sigma_y = 10^{-4}$, $\hat{\sigma}_x = 0.1$, $\hat{\sigma}_y = 0.1$.

References

1. Gillespie DT. Exact stochastic simulation of coupled chemical reactions. The Journal of Physical Chemistry. 1977;81(25):2340–2361.

2. Gibson MA, Bruck J. Efficient exact stochastic simulation of chemical systems with many species and many channels. The Journal of Physical Chemistry A. 2000;104(9):1876–1889.

3. Anderson DF. A modified next reaction method for simulating chemical systems with time dependent propensities and delays. The Journal of Chemical Physics. 2007;127(21).

4. Cai XD. Exact stochastic simulation of coupled chemical reactions with delays. The Journal of Chemical Physics. 2007;126(12).

5. Barrio M, Burrage K, Leier A, Tian TH. Oscillatory regulation of Hes1: discrete stochastic delay modelling and simulation. PLoS Computational Biology. 2006;2(9):e117.

6. Van Kampen NG. Stochastic Processes in Physics and Chemistry. Elsevier; 1992.

7. Gardner TS, Cantor CR, Collins JJ. Construction of a genetic toggle switch in Escherichia coli. Nature. 2000;403(6767):339–342.

8. Liu CB, Wang J Distilling dynamical knowledge from stochastic reaction networks. Proceedings of the National Academy of Sciences, 2024, 121(14): e2317422121.

9. Tang Y, Weng J, Zhang P. Neural-network solutions to stochastic reaction networks. Nature Machine Intelligence. 2023;5(4):376–385.

10. Terebus A, Liu C, Liang J. Discrete and continuous models of probability flux of switching dynamics: Uncovering stochastic oscillations in a toggle-switch system. The Journal of Chemical Physics. 2019;151(18).
